# Evaluation of focused sentinel lymph node RT-qPCR screening for micrometastases with the use of the Maruyama computer program

**DOI:** 10.1007/s10353-013-0226-8

**Published:** 2013-08-17

**Authors:** T. Jagric, S. Potrc, A. Ivanecz, M. Horvat, M. Plankl, T. Mars

**Affiliations:** 1 Department of Abdominal and General Surgery, University Medical Centre Maribor, Ljubljanska 5, 2000 Maribor, Slovenia; 2 Department of General and Abdominal Surgery, University Clinical Centre Maribor, Ljubljanska 5, 2000 Maribor, Slovenia; 3 Faculty of Medicine, Institute of Pathophysiology, University of Ljubljana, Zaloska 4, 1000 Ljubljana, Slovenia; 4 Faculty of Medicine, University of Maribor, Slomskov Trg 15, 2000 Maribor, Slovenia

**Keywords:** Gastric cancer, Sentinel lymph node, RT-qPCR, Maruyama computer program

## Abstract

**Background:**

In this preliminary study, we investigated the sensitivity and specificity of reverse transcriptase (RT)-qPCR lymph node (LN) metastases detection, the accuracy of intraoperative dye navigation, and the incidence of micrometastasis (MM) detection with this protocol, compared to other published studies.

**Methods:**

A total of 23 patients were enrolled in the study. The first stained LN was analyzed using RT-qPCR for carcinoembryonic antigen (CEA) and cytokeratin 20 (CK-20) expression, as markers for MM involvement. The Maruyama computer program was used to determine the most likely first metastatic site. These results were compared with the actual staining patterns to evaluate whether the first draining LN was extracted. We analyzed the correlations between MM and tumor characteristics. The incidence of MM detected with the present method was compared to other studies, as markers of the accuracy of the present protocol.

**Results:**

At 35 threshold cycles, the RT-qPCR had a negative predictive value of 100 % and a positive predictive value of 83.3 %. MM were detected in 4 out of 14 node-negative patients (28.6 %). The extracted sentinel LN coincided in 76.9 % of cases with the most probable first metastatic LN predicted by the Maruyama program. MM were found more frequently in these ‘high-risk’ LNs. Significant differences were found in the Lauren’s histological type distribution and the age distribution among the MM-positive and MM-negative groups.

**Conclusion:**

Our preliminary results confirm that RT-qPCR is an accurate method of MM detection, that the dye navigation enables the determination of the first draining LN, and that the incidence of MM detection with this focused sentinel LN protocol is comparable to other studies.

## Introduction

Since its first publication by Morton et al. for melanoma patients in 1992 [1, 2], the concept of sentinel lymph node (SLN) has been adopted in different fields of oncological surgery, and most recently in gastric cancer surgery. SLN is defined as the first node that receives the cancer-cell drainage from a primary tumor [3, 4], which leads to the concept that micrometastases (MM) will develop in the SLN first. Although the value of these ‘dormant’ metastases is still awaiting clinical confirmation, numerous studies have indicated the prognostic significance of MM [5–7].

The controversy of this subject has been fuelled by the varying results in published papers. Many studies have indicated that isolated tumor cells in lymph nodes (LNs) lack the proliferative capacity to develop full-blown LN metastasis [3, 8, 9]. However, a primary reason behind the inability to push such a concept into clinical practice is the method for MM detection. Many studies have used routine histological staining with hematoxylin and eosin, which lacks sensitivity and specificity [10]. Immunohistochemical staining has been reported to improve sensitivity and to be reliable, although this can generate false negative results by overlooking possible MM that are localized outside the cut slice, or false positive results due to antibody cross-reactivity with host stromal or inflammatory cells [10, 25]. It has been reported that reverse transcriptase polymerase chain reaction (RT)-PCR is the most sensitive method for the detection of MM [11]. The drawbacks of this method are the high cost and that it is labor intensive when it is used to evaluate all harvested LNs, which limits its usefulness particularly in the research setting. Therefore, we searched for an alternative method, to retain the benefits of RT-qPCR, while still obtaining results in the time-frame necessary to tailor decisions about the extent of the lymphadenectomy. While the new RT-qPCR protocols allow the shortening of the procedure of LN analysis to 1 h [5, 26], it is still not possible to analyze more than one SLN in such a short period. The only way to reduce this time with the present methods of MM detection is to reduce the number of LNs screened. We modified the present gastric cancer SLN protocols with the definition of a new hypothesis: that tumor cells spread in a predictable fashion, and with correct LN navigation, there is only the need to analyze one ‘high-risk’ SLN to obtain the correct regional LN staging.

To evaluate the adequacy of such a concept, one would have to assess the recurrence or survival rates, which requires a lengthy study period. However, preliminary results can be obtained with the use of the Maruyama computer program. This program predicts the probability of LN metastases of individual gastric cancer patients, by comparing their characteristics with those contained in its database [12]. Although this program is not designed for the prediction of MM, it suggests which nodal stations are most likely to be the first metastatic site. Hence, these sites might, according to the definition, also have the greatest risk of receiving the first isolated tumor cells or MM during tumorigenesis.

Thus, the aim of this preliminary study was to determine the usefulness of this focused SLN protocol, by determining whether the LN first marked with Patent Blue V dye is most likely to be the first metastatic site. We therefore assessed the accuracy of RT-qPCR detection of metastatic cells in the LNs, and determined whether the intraoperative dye navigation helps in defining the most probable first-draining LN. Finally, we compared the incidence of MM detected using the present method with other studies, where different methods were used and more SLNs per patient were analyzed.

## Methods

### Patients

Twenty-three patients who underwent curative resection in the Department for General and Abdominal Surgery at the University Clinical Centre of Maribor (Slovenia) were included in this study, which started in August 2009. Of these 23 patients, 9 were selected as the control group. Five patients who were operated on for a pathology other than gastric cancer were selected as the negative control group (right femoral hernia, benign pyloric stenosis, Crohn’s disease, hepatocellular carcinoma, sigmoid adenoma). The positive control group consisted of four patients with LN-positive gastric cancer. During these operations, the LNs were extracted from the operative field, stored and subsequently analyzed with RT-qPCR in a similar fashion to the study group. In the positive control group, overtly metastatic LNs were extracted for RT-qPCR analysis. The positive and negative control groups were used to determine the sensitivities and specificities of the RT-qPCR LN metastasis detection at different threshold cycles (Ct), and to obtain a Ct for further analysis.

All patients in the study group had histologically verified gastric adenocarcinoma. Preoperative staging was performed. Following this work-up, potential Node Zero (N0) patients were assessed for inclusion in the present study. Only patients who were confirmed to be N0 after formal patho-histological analysis of the surgical specimens were included in the present study. All LN specimens were examined using standard hematoxylin-eosin staining, and tumor staging was performed according to the 7th Edition of the International Union Against Cancer TNM classification. Out of the 19 patients included, 14 were confirmed as N0 after the patho-histological analysis. All the patients were presented to a tumor board for neoadjuvant chemotherapy or adjuvant chemoradiotherapy eligibility. Patients with a preoperative clinical stage of T3 (subserosal tumor), T4 (tumor spread beyond the serosa) or N+ (positive lymph nodes) were considered for neoadjuvant therapy. Patients with a pathological stage of T3, T4a/b or with positive LNs received adjuvant treatment.

All patients gave their written informed consent before being included in the present study. This study was approved by the National Ethics Committee.

### Surgical and sentinel lymph node staining techniques and extraction

Surgery started with the exploration of the abdominal cavity. The tumor site, clinical stage and resectability were defined. Patients with overt nodal metastases or tumors spread beyond the serosa were excluded from the study. The surgery and the patho-histological evaluation were performed according to standards described elsewhere for gastric cancer [13].

**Fig. 1:**
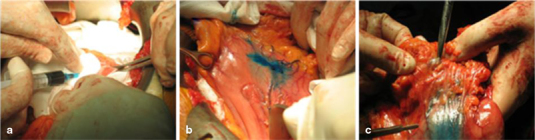
SNL mapping with Patent Blue V dye. **a** Submucosal infiltration with the dye. **b** Identification of lymphangial vessels and drainage to the first LN. **c** Extraction of the first-draining LN

Before the dissection was performed, Patent Blue V Dye (Guerbet Patent Blue V Sodium 2.5 %, Guerbet, Roissy, France) was injected in four to five sectors of the submucosa around the primary tumor (Fig. 1). The lymphatic drainage of the dye was followed for the first tumor-draining LN. After 17 ± 6.8 min, the first LN with the dye was extracted for RT-qPCR analysis. This LN was then immediately placed in RNAlater RNA Stabilization Reagent. The submerged specimens were incubated in this reagent at 0–4 °C, and analyzed within a month.

### mRNA extraction from sentinel lymph nodes and RT-qPCR analysis

The total RNA extracted with RNeasy Mini Plus kits (Qiagen, Hilden, Germany) was reverse transcribed using High Capacity cDNA Reverse Transcription kits (Applied Biosystems). Q-PCR was performed on an ABI PRISM 7500 Sequence Detection System (Applied Biosystems), using TaqMan chemistry in a 96-well format. TaqMan Universal PCR Master Mix (Applied Biosystems) and the following Gene Expression Assays (Applied Biosystems) were used for CEACAM5 Hs 00237075_m1, KRT20 Hs00300643_m1, and GAPDH 4333764. Thirty-five cycles were selected as the Ct threshold values for CEACAM5 and CK-20 expression, as determined elsewhere [4, 10].

## Statistical analysis

Continuous data are expressed as means ± SD, while categorical variables are given as percentages. The Shapiro-Wilk test was used to determine whether the continuous data were normally distributed. Comparisons of continuous variables were carried out with Student’s t-tests for parametric data and Mann–Whitney U test for non-parametric data. Chi-square tests were used for comparisons of discrete variables. The sensitivity and specificity of the MM determinations were evaluated with a two-by-two contingency table, where the expression profiles of positive and negative controls were compared. The Maruyama computer program was used to calculate the probabilities of metastases in 16 LN stations. *P* values < 0.05 were defined as the limit of significance. For statistical analysis, PASW version 18 for Windows 7 was used. The probability of LN involvement was estimated with WinEstimate (version 2.5, München, Germany).

## Results

The patient and tumor characteristics for the study group are given in Table 1. Out of the 12 patients included, eight were N0 patients and had intestinal type of gastric cancer (66.7 %). The predominant location of the tumors was the lower third (nine cases; 69.2 %) and the lesser curvature (five cases; 35.7 %) of the stomach. Early gastric cancer was found in nine patients (57.1 %). Five patients had locally advanced gastric cancer. The cancer was mostly poorly differentiated tubular adenocarcinoma (five cases; 41.7 %). Only two patients from the study group (14.3 %) received chemotherapy. Both of these two patients were under-staged with preoperative diagnostics and had not received neoadjuvant treatment. After the formal pathohistological analysis, one of these two patients was found to have a T4a stage tumor, while the other had a T3 stage tumor. In each case, these patients received adjuvant treatment with capecitabine plus radiotherapy.

**Table 1:** Patient and tumor characteristics

Characteristic	*n*(%) value
*Gender*	
Male	10 (71.4 %)
Female	4 (28.6 %)
Age (years)	65 ± 10.8
*Lauren classification*	
Intestinal	8 (66.7 %)
Diffuse	2 (16.7 %)
Mixed	2 (16.7 %)
*Tumor location*	
Mid third	4 (30.8 %)
Lower third	9 (69.2 %)
*Tumor site*	
Lesser curvature	5 (35.7 %)
Greater curvature	3 (21.4 %)
Anterior wall	1 (7.1 %)
Posterior wall	4 (28.6 %)
Circular involvement	1 (7.1 %)
*Early gastric cancer type*	
I	2 (22.2 %)
IIb	2 (22.2 %)
IIc	3 (33.3 %)
III	2 (22.2 %)
*Bormann type*	
III	3 (60 %)
IV	2 (40 %)
*T stage*	
T1a	5 (35.7 %)
T1b	3 (21.4 %)
T2	4 (28.6 %)
T3	2 (14.3 %)
*Differentiation*	
Tubular well differentiated	3 (25 %)
Tubular moderate differentiated	3 (25 %)
Tubular poor differentiated	5 (41.7 %)
Signet ring-cell	1 (8.3 %)
CEA (ng/ml)	1.4 ± 1.07
AFP (ng/ml)	3.5 ± 1.92
CA 19-9 (ng/ml)	10.33 ± 13.27
Tumor size (mm)	34.1 ± 22.11
Extraction time (min)	17.8 ± 6.9
Total number of resected lymph nodes	12.4 ± 7.9

To evaluate the sensitivity, specificity, and the positive and negative predictive values of RT-qPCR for carcinoembryonic antigen (CEA) and cytokeratin 20 (CK-20) detection, a two-by-two contingency table was constructed (Table 2). The sensitivity of this method at Ct 35 cycles was 75 %, with a specificity of 100 %. The negative predictive value was 100 %, and the positive predictive value was 83.3 %. These results obtained with Ct 35 cycles were comparable to other studies, and therefore the further RT-qPCR analysis was carried out with this threshold value.

**Table 2:** Two-by-two contingency table

	Control		Total
	Negative	Positive	
RT-qPCR negative	4	11	15
RT-qPCR positive	0	7	7
*Total*	*4*	*18*	*22*

The next step was the evaluation of the study group (Table 3). Of the 14 histologically confirmed N0 patients, 4 (28.6 %) showed MM, while no CEA or CK-20 expression was detected in the remaining 10 patients, who were thus MM negative. There were no differences in gender distribution, preoperative tumor marker values, location of tumor, early gastric cancer type, Bormann type, tumor size, number of resected LNs, vascular, lymphangial or perineural invasion, extranodal infiltration, histological grade, and T-stage distribution. The patients in the MM-positive group were significantly older than the patients with no MM (55 ± 8.7 vs. 69 ± 8.9 years; *P* = 0.019). A significant difference was noted in the Lauren histological type distribution (*P* = 0.037). In the MM-negative group, the most prevalent histological type was intestinal, in 88.9 % of cases (eight patients). In the MM-positive group, most of the patients had the diffuse type of gastric cancer (66.7 %; two patients). No patient with MM had an intestinal type of cancer.

**Table 3:** Comparison of MM-positive and MM-negative groups

	RT-qPCR expression (*n*, %)		*P*
	Positive	Negative	
Gender			NS
Male	2 (50 %)	8 (80 %)	
Female	2 (50 %)	2 (20 %)	
Age (years)	55 ± 8.7	69 ± 8.9	0.019
Lauren type			0.037
Intestinal	0 (0 %)	8 (88.9 %)	
Diffuse	2 (66.7 %)	0 (0 %)	
Mixed	1 (33.3 %)	1 (11.1 %)	
Tumor third			NS
Mid third	2 (50 %)	2 (22.2 %)	
Lower third	2 (50 %)	7 (77.8 %)	
Tumor site			NS
Lesser	2 (50 %)	3 (30 %)	
Greater	1 (25 %)	2 (20 %)	
Anterior	0 (0 %)	1 (10 %)	
Posterior	1 (25 %)	3 (30 %)	
Circular	0 (0 %)	1 (10 %)	
EGC type			NS
I	1 (50 %)	1 (14.3 %)	
IIb	0 (0 %)	2 (28.6 %)	
IIc	1 (50 %)	2 (28.6 %)	
III	0 (0 %)	2 (28.6 %)	
Bormann type			NS
III	1 (50 %)	2 (66.7 %)	
IV	1 (50 %)	1 (33.3 %)	
T stage			NS
T1a	1 (25 %)	4 (40 %)	
T1b	1 (25 %)	2 (20 %)	
T2	1 (25 %)	3 (30 %)	
T3	1 (25 %)	1 (10 %)	
Differentiation			NS
Tubular well	0 (0 %)	3 (33.3 %)	
Tubular moderate	1 (33.3 %)	2 (22.2 %)	
Tubular poor	2 (66.7 %)	3 (33.3.%)	
Signet ring-cell	0 (0 %)	1 (11.1 %)	
Lymphangial invasion			NS
Negative	4 (100 %)	9 (90 %)	
Positive	0 (0 %)	1 (10 %)	
Vascular invasion			NS
Negative	4 (100 %)	10 (100 %)	
Positive	0 (0 %)	0 (0 %)	
Perineural invasion			NS
Negative	3 (75 %)	8 (80 %)	
Positive	1 (25 %)	2 (20 %)	
Extranodal infiltration			NS
Negative	4 (100 %)	10 (100 %)	
Positive	0 (0 %)	0 (0 %)	
CEA (ng/ml)	1 ± 0	1.57 ± 1.3	NS
AFP (ng/ml)	3 ± 1.7	3.8 ± 2.2	NS
CA 19-9 (ng/ml)	22 ± 17.8	4 ± 5	NS
Tumor size (mm)	30 ± 12	35 ± 25.5	NS
Number of extracted lymph nodes	13 ± 9.8	12 ± 7.5	NS
Proportion of positive lymph nodes (%)	52 ± 43.7	26 ± 28.2	NS

All of the patients were analyzed retrospectively with the WinEstimate computer program, to estimate the likelihood of LN metastases. With this program, the most likely metastatic site was determined and compared with the LN stations determined to be the first-draining site of the tumor with intraoperative Patent Blue V staining. The extracted LN coincided in 76.9 % with the WinEstimate-predicted LN stations in the N0 patients. Although insignificant due to the low case numbers, MM were more frequently found in correctly predicted nodes (with a 3:1 ratio in favor of the correctly predicted LN).

## Discussion

With a more liberal use of gastroscopy in symptomatic patients, there has been a slow but significant rise in the detection of early gastric cancer in Slovenia [14, 15]. In these early favorable stages, limited and less aggressive treatment methods are being explored; however, these must rely on more accurate tumor staging. Unfortunately, till date, there is no preoperative diagnostic tool that can reliably detect nodal metastases in gastric cancer patients [28]. Usually, surgeons rely on the scarce preoperative staging and the intraoperative clinical assessment to determine the best type of resection for early and node-negative gastric cancer. As the first metastatic deposits in early gastric cancer are usually in the form of MM, such clinical staging during the operation is at best questionable. If a restrictive policy towards extended lymphadenectomy is taken, as many as 20 % of early gastric cancer patients will have an insufficiently defined operation [1]. Therefore, other methods of selection for more extensive operations are being explored.

The SLN concept that has revolutionized melanoma and breast cancer treatment might bring new concepts to gastric cancer surgery. However, in contrast to breast cancer or melanoma, where metastatic deposits follow a predictable path to the LNs and are large enough to be detected in frozen sections, so that the relevant results can be obtained in 30 min [3]. LN metastases in early gastric cancer are usually packed into small groups of cells on the periphery of the lymph node [5]. This makes it harder to detect them with routine histological methods. To increase the sensitivity and specificity for MM detection, extensive sectioning is at present necessary to obtain representative sensitivities and specificities. Many studies even resort to immunohistochemical and molecular analysis of large numbers of lymph nodes [10, 11, 25]. While RT-qPCR is said to be the most sensitive and specific method, it is still notoriously elaborate and expensive; and it takes too long to provide an efficient selection tool for the surgeon. We therefore tried to modify the protocols used at present to detect MM, to retain some of the advantages of this molecular tool while reducing the time to obtain relevant results. The only way to achieve such an endeavor was to reduce the number of LNs examined, by examining only the first-draining SLN.

To determine the validity of the proposed focused SNL protocol long-term survival analysis is necessary. Patients with early gastric cancer have excellent survival [1], so the first impact of MM is expected to be on disease-free survival of the patients. When present in early gastric cancer, the first signs of local failure develop after 5 years [20]. As the first relevant results can only be expected after such a long observational period, we designed a preliminary study to determine whether MM can be reliably detected by examination of only one ‘high-risk’ SLN. This is, in our opinion, a critical prerequisite before embarking on a larger trial for the clinical application of this concept. We therefore investigated the sensitivity of RT-qPCR detection of LN metastases, the accuracy of the intraoperative dye navigation, and the incidence of MM detection using this protocol, as compared to other published studies.

The double marker RT-qPCR assays used in the present study proved to be a sensitive and specific tool for the detection of MM in LNs. At Ct 35 cycles, we achieved a negative predictive value of 100 % and a positive predictive value of 83.3 % in the control groups. These values confirm the possibility for the confident use of RT-qPCR for further investigations. Similar results were obtained in other studies, where RT-qPCR was determined to have superior sensitivities and specificities to standard hematoxylin and eosin analysis and immunohistochemical staining [4, 5, 10, 16].

Even if we achieve impressive accuracies with these RT-qPCR double-marker assays, the method in itself does not allow the time interval of the analysis to be shortened when all of the LNs from a stained LN basin are screened for MM. In similar studies, up to 15 LNs were examined with a mean of 2–4 [3–10, 16–19]. The present study is thus in sharp contrast here, with only one examined SLN. We based our protocol on extensive lymphograpy studies, which have shown that at least for early gastric cancer, MM occur according to a predictable path [16, 27]. Indeed, even in studies where extensive immunohistochemical staining analysis of LNs of the pregastric compartment were performed, MM were never found only outside the stained SLNs [19]. It can be assumed that in spite of a more complex lymphatic drainage of the stomach wall, MM are usually first formed in the SLNs. It would thus theoretically suffice to examine only the first-draining node, under the condition that the extracted LN is indeed the true SLN.

To determine whether dye navigation can reliably define the most-probable first-draining site, the Maruyama computer program was used. The extracted LNs in the present study coincided in 76.9 % of cases with the nodal station predicted to be the most frequent metastatic site by the Maruyama computer program. We also determined that although not reaching significance in the present study, MM were more frequent in the nodes predicted by the Maruyama program to be the most-probable first metastatic site. Not only does this confirm that dye navigation leads to the correct definition of the first-draining node, but it also emphasizes the importance of the correct LN navigation. Meticulous intraoperative dye injections as close as possible to the tumor are of the utmost importance for the present method. Perhaps additional methods, such as the preoperative labeling of the tumor site with dyes containing particles of a specific size that will remain lodged only in the first-draining node [5], can be used to increase the yield of ‘true’ SLNs. The evolution of such staining patterns remains a subject of future studies.

Even if we extract the first-draining node, it still does not confirm that MM can be reliably detected. The present method can still suffer from insufficient sensitivities of RT-qPCR for MM detection, aberrant marker expression, and inadequate staining patterns. An indication for appropriate MM detection is the MM frequency. If MM are missed because of the analysis of the wrong SLN, a much lower incidence of MM would be expected than in comparable studies, where more LNs per patient are examined. In our cohort, 4 out of 12 node-negative patients (28.6 %) were MM positive. In other studies, the presence of MM ranged from 10 to 30 % [3–10, 16–19].

Our results also indicate that MM are more common in older patients and in the diffuse type of gastric cancer. The higher prevalence of MM in the older population might reflect their lower immunocompetence. Sansoni et al. reported dysfunction of the natural killer cells in older patients, which can fail to clear isolated tumor cells lodged in LNs, resulting in higher incidence of MM [3, 20, 21]. Higher incidence of MM in the diffuse type of gastric cancer has also been reported in other studies [22]. These findings can be extremely important in the work-up of preoperative patients, and they need to be further explored in future studies.

Taken together, our preliminary results confirm that RT-qPCR is an accurate method of MM detection, that dye navigation enables the first-draining node to be found, and analysis of the ‘high-risk’ SLN can detect MM with the same efficiency as the more elaborate LN screening protocols. Considering these findings, we are determined to refine our method so as to more fully merge the advantages of the superior sensitivities and specificities of RT-qPCR with the cost benefits and the speed of this focused SLN concept.

## Conclusions

Although these data indicate that single SLN screening is an accurate method of SLN evaluation, these results remain preliminary. Only recurrence and long-term survival analysis on larger series of patients will be able to reveal the full validity of the present method. If this method is further proven to be effective, the results can be used intraoperatively. With the development of methods such as transcription-reverse transcription concerted reaction, one-step nucleic-acid amplification assay, and fully automated multiplex quantitative RT-PCR, which generate results that are equal to standard RT-qPCR in no more than 20 min to 1 h [5, 26], single SLN analysis might be the first step along the road toward intraoperative decision making, which would thus usher in the age of tailored, limited surgery for gastric cancer.

### Conflict of interest

This study was supported by a national ARRS grant. With this statement, the authors declare that there are no conflicts of interest.
